# On the Cure of Epilepsy by Ligature of the Vertebral Arteries

**Published:** 1883-02

**Authors:** 


On the Cure of Epilepsy by Ligature of the Vertebral Arteries.—Dr. Alexander recounts twenty-two cases of epilepsy where ligature of the vertebral arteries was practiced. Three patients remained unattacked for a year, nine were but recently operated on—these, however, have had no recurrence as yet; eight show decided improvement; and one died in a convulsion, two months subsequent to the ligature.

